# Ligand engagement of Toll-like receptors regulates their expression in cortical microglia and astrocytes

**DOI:** 10.1186/s12974-015-0458-6

**Published:** 2015-12-30

**Authors:** Carla Marinelli, Rosa Di Liddo, Laura Facci, Thomas Bertalot, Maria Teresa Conconi, Morena Zusso, Stephen D. Skaper, Pietro Giusti

**Affiliations:** Department of Pharmaceutical and Pharmacological Sciences, University of Padua, Largo “E. Meneghetti” 2, 35131 Padua, Italy

**Keywords:** Microglia, Astrocyte, Toll-like receptor, Cytokine, Nuclear factor-κB, Neuroinflammation

## Abstract

**Background:**

Toll-like receptor (TLR) activation on microglia and astrocytes are key elements in neuroinflammation which accompanies a number of neurological disorders. While TLR activation on glia is well-established to up-regulate pro-inflammatory mediator expression, much less is known about how ligand engagement of one TLR may affect expression of other TLRs on microglia and astrocytes.

**Methods:**

In the present study, we evaluated the effects of agonists for TLR2 (zymosan), TLR3 (polyinosinic-polycytidylic acid (poly(I:C)), a synthetic analogue of double-stranded RNA) and TLR4 (lipopolysaccaride (LPS)) in influencing expression of their cognate receptor as well as that of the other TLRs in cultures of rat cortical purified microglia (>99.5 %) and nominally microglia-free astrocytes. Elimination of residual microglia (a common contaminant of astrocyte cultures) was achieved by incubation with the lysosomotropic agent l-leucyl-l-leucine methyl ester (L-LME).

**Results:**

Flow cytometric analysis confirmed the purity (essentially 100 %) of the obtained microglia, and up to 5 % microglia contamination of astrocytes. L-LME treatment effectively removed microglia from the latter (real-time polymerase chain reaction). The three TLR ligands robustly up-regulated gene expression for pro-inflammatory markers (interleukin-1 and interleukin-6, tumor necrosis factor) in microglia and enriched, but not purified, astrocytes, confirming cellular functionality. LPS, zymosan and poly(I:C) all down-regulated TLR4 messenger RNA (mRNA) and up-regulated TLR2 mRNA at 6 and 24 h. In spite of their inability to elaborate pro-inflammatory mediator output, the nominally microglia-free astrocytes (>99 % purity) also showed similar behaviours to those of microglia, as well as changes in TLR3 gene expression. LPS interaction with TLR4 activates downstream mitogen-activated protein kinase and nuclear factor-κB signalling pathways and subsequently causes inflammatory mediator production. The effects of LPS on TLR2 mRNA in both cell populations were antagonized by a nuclear factor-κB inhibitor.

**Conclusions:**

TLR2 and TLR4 activation in particular, in concert with microglia and astrocytes, comprise key elements in the initiation and maintenance of neuropathic pain. The finding that both homologous (zymosan) and heterologous (LPS, poly(I:C)) TLR ligands are capable of regulating TLR2 gene expression, in particular, may have important implications in understanding the relative contributions of different TLRs in neurological disorders associated with neuroinflammation.

**Electronic supplementary material:**

The online version of this article (doi:10.1186/s12974-015-0458-6) contains supplementary material, which is available to authorized users.

## Background

Inflammation is the body’s attempt at self-protection to remove harmful stimuli, including damaged cells, irritants and pathogens—and initiate the healing process. Activation of the innate immune system is an integral aspect of the response to inflammation. When prolonged, however, inflammation can become adversarial. Inflammation and neurological diseases are intimately connected, with ever-growing evidence pointing to its being a key feature in the pathobiology of neuropathic pain, chronic neurodegenerative diseases, brain ischemia, spinal cord injury, traumatic brain injury, some neuropsychiatric disorders [1–5] and possibly even autism spectrum disorder [6]. Microglia, the resident macrophage population in the CNS—along with astrocytes—constitute principal players in neuroinflammatory responses [7–13].

The innate immune system relies on a set of germ-line encoded receptors that recognize conserved molecular patterns found only in microorganisms. This large family of so-called pattern recognition receptors includes the Toll-like receptors (TLRs), considered as crucial environmental-sensing molecular motifs for pathogen-associated molecular patterns, which are linked with microbial pathogens or cell stress, as well as danger-associated molecular patterns released during cell damage [14–17]. Rodent microglia express mRNA for all of the recently identified TLRs, TLR1-9, used for recognition of bacterial and viral molecular patterns, whereas other neural cells (e.g. astrocytes) express a more limited TLR repertoire [15, 18]. TLR signalling pathways have been implicated in neurodegenerative disorders [19], including motor neuron disease [20], as well as in pathological pain [21–24].

Recent studies indicate that microglia-free astrocytes, unlike microglia, are unresponsive to TLR engagement in terms of pro-inflammatory molecule output [7, 8, 25]. Further, several of these reports have suggested that microglial cells express a heightened responsivity to TLR agonists when cultured in the presence of astrocytes [7, 8]. While the literature contains numerous examples of astrocyte response to TLR activation, these cell preparations more often than not contain up to 5 % contaminating microglia (e.g. [26]), which can confound the effects observed [27–29]. However, whether or not these observations extend also to the ability of glia TLRs to regulate their own expression, or that of other TLR subtypes, remains to be fully explored. Given that multiple TLRs may be activated in neuropathological settings, the up- or down-regulation of one TLR consequent to engagement of another may have important disease implications. In order to address this question, we interrogated purified cortical microglia and astrocytes for their capacity to express TLR2, TLR3 and TLR4 mRNA and protein upon treatment with either the cognate ligand or that of one of the other TLRs. Our results show a complex pattern of TLR regulation in microglia and, in addition, the capacity of nominally microglia-free astrocytes to both express and respond to TLR agonists in a nuclear factor-κB (NF-κB)-dependent manner.

## Methods

Tissue culture media, antibiotics, fetal calf serum (FCS) and NP40 cell lysis buffer (10×) were purchased from Life Technologies (San Giuliano Milanese, Italy); lipopolysaccaride (LPS) (Ultra-Pure LPS-EB from *E. coli* 0111:B4 strain), zymosan, Pam3CSK4 (VacciGrade™) and polyinosinic-polycytidylic acid (poly(I:C)) (high molecular weight) were from InvivoGen (Cayla-Invivogen Europe, Toulouse, France); BD CytoFix/CytoPerm and CytoFix were from BD Biosciences (SACCO srl, Cadorago (CO), Italy); Ro-106-9920 (Tocris-Cookson, Space Import–export srl, Milan, Italy); poly-l-lysine hydrobromide (mol wt 70,000–150,000), papain, DNase I (bovine pancreas), trypsin inhibitor, l-leucyl-l-leucine methyl ester (L-LME), SB202190, protease inhibitor cocktail, Pefabloc® SC (100 mM), LPS from *E. coli* 026:B6 (<5 % protein impurities), polymyxin B and all other biochemicals were purchased from Sigma-Aldrich (Milan, Italy) unless noted otherwise; Falcon tissue culture plasticware was purchased from BD Biosciences. Sterilin petri plastic dishes (10 cm Ø) were obtained from Sarstedt (Verona, Italy).

### Cell culture

Mixed glial cell cultures from cortex were prepared from postnatal day 1–2 rat pups (strain: CD) as described previously [30]. Experiments were performed in accordance with the National Institutes of Health guidelines for the care and use of laboratory animals and those of the Italian Ministry of Health (D.L. 116/92), and were approved by the Institutional Animal Care and Use Committee. Tissue dissociates were plated in 75-cm^2^ poly-l-lysine-coated tissue culture flasks at a density of 1.5 brains per flask and grown in high-glucose Dulbecco’s modified Eagle’s medium (DMEM) with 2 mM glutamine, 100 units/ml penicillin/50 μg/ml streptomycin, 50 μg/ml gentamicin and 10 % (vol/vol) FCS. Culture medium was changed after 24 h. Upon reaching confluency (approximately 7 days later), microglia were dislodged by shaking the flasks at 200 rpm for 1 h (37 °C). The culture supernatants enriched in microglia were transferred to plastic Petri dishes (Sterilin) and incubated for 45 min at 37 °C (5 % CO_2_, 95 % air) to allow differential adhesion of microglia. Adherent microglia (>99.9 % purity) were mechanically scraped into culture medium and replated in this medium on poly-l-lysine-coated 96-well microwell culture plates or 24-well multiwall plates (50,000 and 250,000 cells per well, respectively). The remaining cell monolayers were highly enriched in astrocytes (<5 % microglia, flow cytometry using cell type-specific antibodies). For some experiments, the astrocyte monolayers were depleted of residual microglia using a 60-min exposure (50 mM) to the lysosomotropic agent L-LME [27], as described previously [7, 8]. Astrocyte plating densities were the same as used for microglia.

### Culture treatments

Microglia or astrocyte cultures were treated with one of the following TLR ligands: 100 ng/ml LPS-EB Ultra-Pure ('LPS') (a selective agonist for TLR4 with no LPS-independent activity); 10 μg/ml zymosan (TLR2 agonist) [31]; 50 μg/ml poly(I:C) (a synthetic analogue of double-stranded RNA which activates TLR3) [32]. Agonists were added in DMEM + 10 % FCS. Cells were treated for 6 or 24 h for gene expression and flow cytometry (FCM) analysis.

### FCM

Monolayers of purified cortical microglia and astrocytes (±L-LME treatment) were washed with phosphate-buffered saline (PBS), scraped into PBS and the cells pelleted by centrifugation (200*g*, 5 min). Samples were fixed with BD CytoFix or fixed/permeabilized with BD CytoFix/CytoPerm at 4 °C for 20 min, depending on whether the antigen of interest was located on the cell surface or intracellularly. Purified microglia and enriched astrocytes were immunophenotypically characterized by FCM using the following primary antibodies against rat markers: Alexa Fluor® 647 mouse anti-glial fibrillary acidic protein (GFAP) (Cell Signaling Technology Europe, Leiden, The Netherlands); rabbit anti-ionized calcium-binding adapter molecule 1 (Iba1) (Wako, Richmond, VA, USA); rabbit anti-TLR2 (sc-10739) polyclonal antibody raised against amino acids 180–354 of TLR2 of human origin (Santa Cruz Biotechnology, Heidelberg, Germany); rabbit anti-rat TLR3 (sc-28999) polyclonal antibody raised against amino acids 26–325 mapping within an N-terminal extracellular domain of TLR4 of mouse origin (Santa Cruz); rabbit anti-rat TLR4 (sc-30002) polyclonal antibody raised against amino acids 339–638 mapping within an N-terminal extracellular domain of TLR4 of mouse origin (Santa Cruz); AlexaFluor® 647 mouse monoclonal antibody IgG1 isotype control (Cell Signaling); AlexaFluor®488 anti-rabbit or anti-mouse secondary antibodies (II Ab) (Life Technologies). For staining, 200,000 cells were incubated with the selected antibody in PBS containing 0.5 % bovine serum albumin (BSA) (Sigma-Aldrich) for 15 min at room temperature. For indirect labelling, the samples were washed with 0.5 % BSA solution and then stained with appropriate secondary antibody. In parallel, samples labelled with isotype or secondary conjugated antibodies were prepared as negative controls. Data were acquired using FACSCanto II Flow cytometer (BD Biosciences) and then analysed with Summit 4.3 (DAKO-Beckman Coulter) and FACSDiva v6.1.3 (BD Biosciences) softwares. The expression of GFAP and Iba1 was reported as geometric mean fluorescent intensity (MFI) ± standard deviation (SD) and percentage of positive cells while data of TLRs were expressed as the ratio of relative MFI (rMFI) values derived from resting (Ctr + 10 % FBS) and primed (+TLR agonist) cultures for each TLR normalized to its II AB-matched negative control (relative MFI). Assuming that a ratio equal to 1 was observed in the case of undetectable difference in primed cells compared to resting samples, values greater or less than 1 were indicative of an increase or decrease in TLR expression, respectively, in samples treated with TLR agonists.

### Quantitative Real-Time-PCR (qRT-PCR)

Total RNA was extracted from cells using the ReliaPrep™ RNA Cell Miniprep System (Promega), according to the manufacturer’s instructions. RNA integrity and quantity were determined by RNA 6000 Nano assay in an Agilent BioAnalyser. Samples were reverse transcribed with Superscript III reverse transcriptase (Life Technologies). The RT-PCR reaction was performed as described previously using a MX 3000P (Stratagene) [7]. Primer sequences are listed in Table 1. Amounts of each gene product were calculated using linear regression analysis from standard curves, demonstrating amplification efficiencies ranging from 90 to 100 %. Dissociation curves were generated for each primer pair showing single product amplification. In the figures, the term ‘fold-increase’ is defined as the cDNA ratio between target gene and reference gene (GAPDH) normalized to untreated control.

**Table 1:** Primer pairs used in this study

Gene target	Primer name	Sequence
GAPDH	GAPDH For	5′-CAAGGTCATCCATGACAACTTTG-3′
	GAPDH Rev	5′-GGGCCATCCACAGTCTTCTG-3′
IL-1ß	IL-1ß For	5′-TGTGGCAGCTACCTATGTCT-3′
	IL-1ß Rev	5′-GGGAACATCACACACTAGCA-3′
TNF-α	TNF-a For	5′-CATCTTCTCAAAACTCGAGTGACAA-3′
	TNF-a Rev	5′-TGGGAGTAGATAAGGTACAGCCC-3′
IL-6	IL-6 For	5′-TCACAGAAGGAGTGGCTAAGG-3′
	IL-6 Rev	5′-GCTTAGGCATAGCACACTAGG-3′
TLR2	TLR2 For	5′-TCCATGTCCTGGTTGACTGG-3′
	TLR2 Rev	5′-AGGAGAAGGGCACAGCAGAC-3′
TLR4	TLR4 For	5′-GATTGCTCAGACATGGCAGTTTC-3′
	TLR4 Rev	5′-CACTCGAGGTAGGTGTTTCTGCTAA-3′
TLR3	TLR3 For	5′-TGAAAACTACGGCGATGCAG-3′
	TLR3 Rev	5′-AGGCAGTTTTACTTCCCCGA-3′

### Western blots

Cell monolayers were washed with cold PBS and to each well was then added 40 μl of lysis solution (890 μl NP40 cell lysis buffer (Life Technologies, 100 μl protease inhibitor cocktail (Sigma-Aldrich), 10 μl of 0.1 M Pefabloc SC (Fluka)). After 30 min on ice, the lysates were collected and cleared by centrifugation at 13,000 rpm for 10 min (Microfuge® 22R centrifuge, Beckman Coulter). The supernatants were retained and stored at −80 °C. Protein content of lysates was determined using the BCA Protein Assay Reagent Kit (Pierce) following the manufacturer’s protocol. Protein samples (10 μg) were separated on a Mini-PROTEAN® Precast Gel (Biorad) with a 4–15 % gradient for 90 min at 140 V. Proteins were electrophoretically transferred onto polyvinylidene difluoride (Merck Millipore) membranes overnight at 4 °C at 25 V. Membranes were then blocked with 3 % BSA and incubated overnight at 4 °C with one of the following primary antibodies: mouse monoclonal against β-actin (working dilution 1:25000; Sigma-Aldrich); rabbit polyclonal against rat TLR4 (working dilution 1:300; Santa Cruz Biotechnology); rabbit polyclonal against rat CD14 (working dilution 1:200; Santa Cruz Biotechnology); rabbit polyclonal against rat MD2 (working dilution 1:1000; Abcam®). The membranes were then washed and incubated 1 h with the appropriate secondary antibody (goat anti-rabbit IgG horseradish peroxidase-conjugated or goat anti-mouse IgG horseradish peroxidase-conjugated, Merck Millipore) diluted 1:4000. Blots were developed using an enhanced chemiluminescence substrate (Sigma-Aldrich) and immunoreactivity visualized utilizing the VersaDoc Imaging System. Protein expression was normalized to β-actin for band density quantification.

### Immunofluorescence

Enriched astrocytes were seeded on poly-l-lysine-coated 12-mm diameter cover glasses (Menzel-Gläser, Menzel GmbH, Germany) placed in the wells of a 12-well plate (500,000 cells per well) and allowed to adhere overnight. The next day, the cells were treated with 50 mM L-LME for 1 h and allowed to recover for 1 day in L-LME-free medium. Cells were fixed with 4 % paraformaldehyde (Sigma-Aldrich), at 4 °C for 30 min. After fixation, cells were washed 3 × 10 min in PBS, pH 7.4. Cells were then permeabilized and blocked with PBS/0.05 % Triton X-100/10 % normal goat serum for 1 h at room temperature, after which time they were incubated overnight with one of the following primary antibodies: mouse monoclonal anti-GFAP antibody (1:400 dilution, Sigma-Aldrich), rabbit polyclonal anti-TLR4 antibody (1:200 dilution, Santa Cruz), or LPS conjugated with Alexa Fluor® 488 (1:200 dilution, Wako, Japan). Cells were washed 3 × 10 min with PBS and subsequently incubated for 1 h at room temperature with Alexa Fluor® 488 goat anti-rabbit IgG or Alexa Fluor® 555 goat anti-mouse IgG secondary antibody (1:500). Nuclei where visualized by incubating for 2 min with DAPI (Boehringer Mannheim, Germany). Cover glasses were mounted onto glass slides using Fluoromount-G (Southern Biotech, USA), and images were acquired on a Leica DMI4000 B microscope equipped for immunofluorescence (Leica Microsystems GmbH, Wetzlar, Germany) using a Leica DFC 480 digital camera (Leica Microsystems GmbH, Wetzlar, Germany).

### Cytokine ELISA assays

Cells were stimulated to release pro-inflammatory mediators in medium containing TLR ligand as indicated in the respective figure legend. Cell supernatants were collected and stored at −20 °C until the day of assay. Cell lysates were prepared as described previously [8]. Interleukin-1β (IL-1β), IL-6 and tumor necrosis factor-α (TNF-α) released into the culture medium (and IL-1β in cell lysates) were analysed using commercially available ELISA kits according to the manufacturer’s instructions (Antigenix America, Huntington Station, NY, USA). Standards with known amounts of IL-1β, IL-6 or TNF-α were used to convert values into absolute concentrations of cytokine in pg/culture well.

### Statistical analysis

Data are given as mean ± SEM. Statistical analyses to determine group differences were performed by one-way analysis of variance, followed by Dunnett’s or Bonferroni’s post-hoc test for comparisons involving more than two data groups. Significance was taken at *p* < 0.05.

## Results

### Flow cytometric analysis of glia cell populations from cortex

**Fig. 1:**
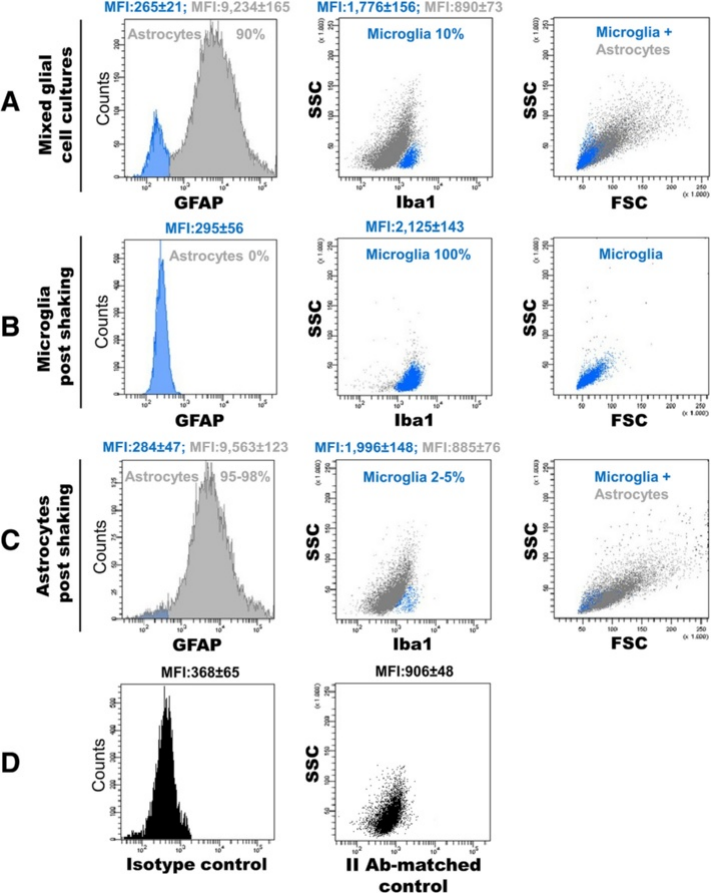
Immunophenotypical characterization by FCM of mixed glial cultures (**a**), microglia (**b**) and astrocytes (**c**) purified by mechanical dislodgment and differential attachment. Data are expressed as percentage (%) of positives ± SD and mean fluorescent intensity (MFI) ± SD for each marker compared to the corresponding isotype or II AB-matched negative sample (**d**). Microglia and astrocytes were further characterized by cell size (forward scatter, FSC) and granularity (side scatter, SSC)

By flow cytometric analysis, mixed glial cell cultures were seen to be enriched in astrocytes as demonstrated by the high expression level of GFAP and large cell size (forward scatter, FSC) (Fig. 1a, left and right panels, respectively). A minimal contamination of 10 % microglia was discriminated based on positive expression of Iba1 marker (Fig. 1a, middle panel) and lower value of cell size (FSC) and granularity (side scatter, SSC) (Fig. 1c). Mechanical dislodgment by plate shaking was efficacious to obtain a population of highly purified cells expressing Iba1 (essentially 100 %) but not GFAP (0 %) (Fig. 1b, middle and left panels, respectively) while the cultures of post-shaken (‘enriched’) astrocytes contained >95 % GFAP^+^ cells with a residual (2–5 %) subset of Iba1^+^ cells (Fig. 1c, left and middle panels, respectively). The positivity of samples for GFAP and Iba1 expression was assessed by comparing the fluorescence intensity of experimental antibody with that of isotype or II Ab-matched negative controls (Fig. 1d).

### TLR agonist-dependent pro-inflammatory profile of purified microglia

**Fig. 2:**
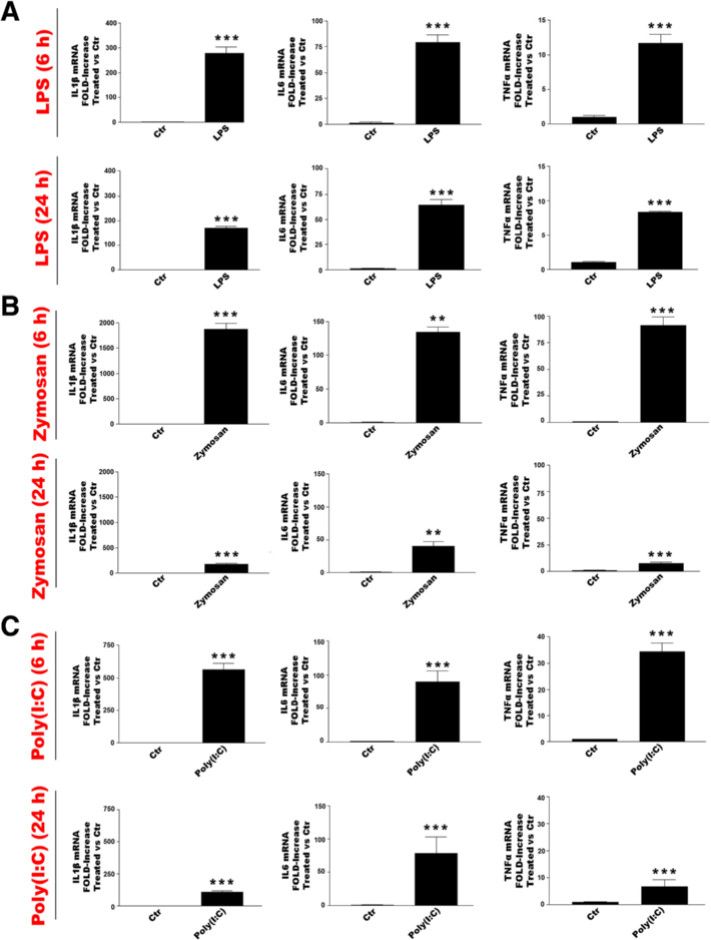
TLR agonists up-regulate pro-inflammatory cytokine gene expression at 6 and 24 h in purified rat cortical microglia. Cells were challenged with **a** LPS (100 ng/ml); **b** zymosan (10 μg/ml); **c** poly(I:C) (50 μg/ml) and processed after 6 and 24 h for IL-1β, IL-6 and TNF-α mRNA expression by RT-PCR. Data are means ± SEM (triplicate culture wells) normalized to GAPDH levels and are representative of three experiments. **p < 0.01 and ***p < 0.001 vs control (‘Ctr’)

Rat cortical microglia responded to TLR2, TLR3 and TLR4 ligands (LPS, zymosan and (poly(I:C)), respectively) after 6 h with a robust up-regulation of mRNA for the pro-inflammatory cytokines IL-1β, IL-6 and TNF-α (Fig. 2a–c, left columns). These mRNA levels remained elevated at 24 h, albeit reduced compared to 6 h (Fig. 2a–c, right columns). Changes in gene expression were accompanied by the corresponding cytokine product in the culture medium at both 6 and 24 h (Tables 2, 3 and 4). The results confirm cellular functionality (see also [7, 18]). Zymosan depleted is a *Saccharomyces cerevisiae* cell wall preparation treated with hot alkali to remove all its TLR-stimulating properties (InvivoGen). Zymosan depleted activates Dectin-1 (which is also expressed in microglia) but not TLR2. Zymosan depleted, up to a concentration of 100 μg/ml, failed to elicit cytokine gene changes or protein release from microglia (data not shown).

**Table 2:** TLR agonists up-regulate TNF-α release at 6 and 24 h in purified rat cortical microglia and enriched, but not microglia-depleted, astrocytes

Treatment	Microglia (pg TNF-α/well)		Astrocytes (pg TNF-α/well)			
			- - -	L-LME	- - -	L-LME
	6 h	24 h	6 h	6 h	24 h	24 h
Control	0	0	0	0	0	0
LPS	272.4 ± 5.7	243.6 ± 3.0	469.4 ± 4.3	6.7 ± 6.7	378.0 ± 2.4	0
Zymosan	307.7 ± 8.9	303.5 ± 5.6	607.4 ± 25.6	34.6 ± 6.9	622.0 ± 89.1	15.7 ± 7.8
Poly(I:C)	209.3 ± 7.2	195.3 ± 10.0	287.4 ± 8.4	11.2 ± 2.4	216.7 ± 4.4	0

Purified microglia and enriched or purified (L-LME-treated) astrocytes were challenged with LPS (100 ng/ml), zymosan (10 μg/ml) or poly(I:C) (50 μg/ml) and processed after 6 and 24 h for TNF-α release into the culture medium by ELISA. Data are means ± SEM (*n* = 3). Note the lack of astrocyte responsiveness after L-LME

**Table 3:** TLR agonists up-regulate IL-1β intracellular content at 6 and 24 h in purified rat cortical microglia and enriched, but not microglia-depleted, astrocytes

Treatment	Microglia (pg IL-1β/well)		Astrocytes (pg IL-1β/well)			
			- - -	L-LME	- - -	L-LME
	6 h	24 h	6 h	6 h	24 h	24 h
Control	16.0 ± 1.0	0	62.6 ± 4.1	37.6 ± 1.3	66.4 ± 2.0	41.5 ± 2.0
LPS	1711.7 ± 63.9	878.1 ± 30.2	5150.4 ± 162.4	154.4 ± 26.7	6636.9 ± 285.5	155.0 ± 17.6
Zymosan	2036.2 ± 53.9	1382.6 ± 29.1	6910.8 ± 742.0	186.5 ± 15.8	7081.9 ± 405.1	157.3 ± 15.2
Poly(I:C)	1710.0 ± 125.1	631.1 ± 41.7	3911.5 ± 96.8	138.5 ± 4.3	3174.6 ± 151.7	62.6 ± 0.7

Purified microglia and enriched or purified (L-LME-treated) astrocytes were challenged with LPS (100 ng/ml), zymosan (10 μg/ml) or poly(I:C) (50 μg/ml) and processed after 6 and 24 h for intracellular IL-1β content by ELISA. Data are means ± SEM (*n* = 3). Note the lack of astrocyte responsiveness after L-LME. Values for IL-1β content of the corresponding culture media samples were <5 % of the intracellular values (not shown)

**Table 4:** TLR agonists up-regulate IL-6 release at 6 and 24 h in purified rat cortical microglia and enriched, but not microglia-depleted, astrocytes

Treatment	Microglia (pg IL-6/well)		Astrocytes (pg IL-6/well)			
			- - -	L-LME	- - -	L-LME
	6 h	24 h	6 h	6 h	24 h	24 h
Control	0	0	0	0	3.5 ± 3.5	0
LPS	26.3 ± 3.1	34.4 ± 0.2	75.5 ± 3.1	0	123.0 ± 1.6	4.3 ± 2.2
Zymosan	29.3 ± 0.8	64.5 ± 4.3	90.4 ± 3.5	0	130.3 ± 4.3	5.5 ± 2.9
Poly(I:C)	12.0 ± 1.5	23.4 ± 2.3	49.7 ± 2.0	0	79.1 ± 3.6	7.7 ± 4.6

Purified microglia and enriched or purified (L-LME-treated) astrocytes were challenged with LPS (100 ng/ml), zymosan (10 μg/ml) or poly(I:C) (50 μg/ml) and processed after 6 and 24 h for IL-6 release into the culture medium by ELISA. Data are means ± SEM (*n* = 3). Note the lack of astrocyte responsiveness after L-LME.

### TLR agonist-dependent pro-inflammatory profile of enriched and purified astrocytes

**Fig. 3:**
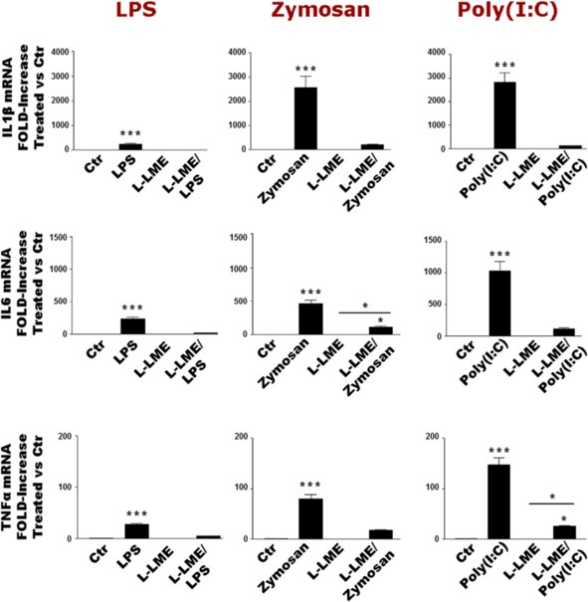
TLR agonists up-regulate pro-inflammatory cytokine gene expression in enriched but not in purified rat cortical astrocytes. Enriched or purified (L-LME-treated) astrocytes were challenged with LPS (100 ng/ml), zymosan (10 μg/ml) or poly(I:C) (50 μg/ml) and processed after 6 h for IL-1β, IL-6 and TNF-α mRNA expression by RT-PCR. Data are means ± SEM (*n* = 3) normalized to GAPDH levels and are representative of three experiments. *p < 0.05 and ***p < 0.001 vs control (‘Ctr’). For L-LME-treated cultures, significant differences between the ‘L-LME’ and ‘L-LME/agonist’ groups were noted only in two cases, as indicated by the bar (*p < 0.05). Qualitatively similar results were obtained after 24 h, although relative mRNA levels were lower

Rodent primary astrocyte cell cultures prepared by standard protocols generally contain variable, small percentages (up to 5 %) of contaminating microglia [28]. As inflammatory mediator output from enriched astrocytes is dependent on the presence of residual microglia [7, 8, 25, 29], we used the lysosomotropic agent L-LME [33] to deplete the remaining microglia [7, 8, 27, 34, 35] (Additional file 1: Figure S1). While LPS, zymosan and poly(I:C) markedly up-regulated mRNA for IL-1β, IL-6 and TNF-α after 6 h, parallel L-LME-treated cultures showed little, if any, response (Fig. 3). Changes in gene expression were again accompanied by increased amounts of the corresponding cytokine product in the culture medium (or intracellularly, in the case of IL-1β), with L-LME-treated astrocytes showing very little output (Tables 2, 3 and 4) (see also [7, 8]).

### TLR agonists effect TLR subtype gene expression in microglia

**Fig. 4:**
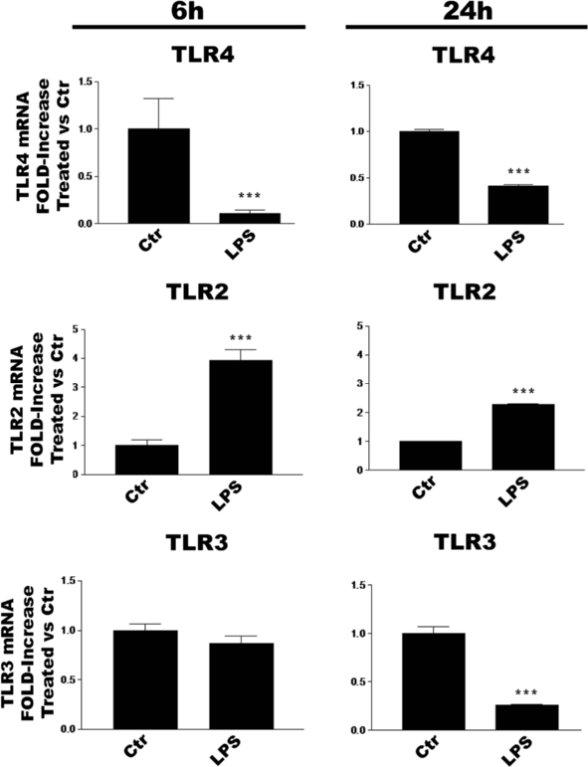
LPS regulates TLR2, TLR3 and TLR4 mRNA expression in rat cortical microglia. Cells were incubated with 100 ng/ml LPS for 6 or 24 h and then processed for RT-PCR analysis. Data are means ± SEM (*n* = 3) normalized to GAPDH levels and are representative of three experiments. ***p < 0.001 vs control (‘Ctr’)

**Fig. 5:**
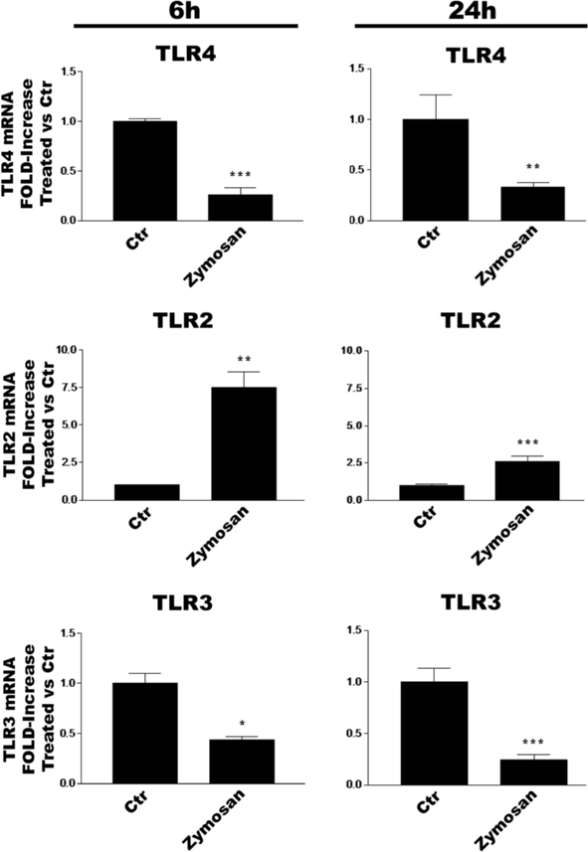
Zymosan regulates TLR2, TLR3 and TLR4 mRNA expression in rat cortical microglia. Cells were incubated with 10 μg/ml zymosan for 6 or 24 h and then processed for RT-PCR analysis. Data are means ± SEM (*n* = 3) normalized to GAPDH levels and are representative of three experiments. *p < 0.05, **p < 0.01 and ***p < 0.001 *vs* control (‘Ctr’)

**Fig. 6:**
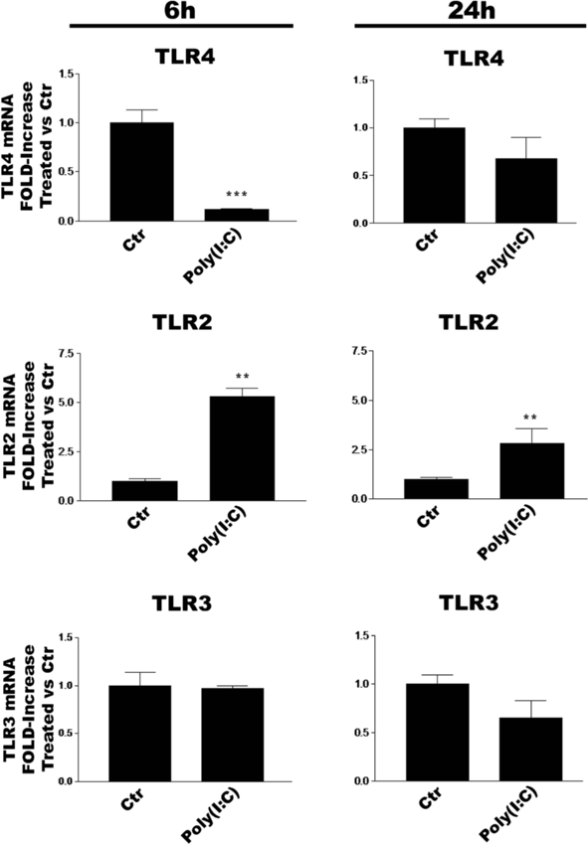
Poly(I:C) regulates TLR2, TLR3 and TLR4 mRNA expression in rat cortical microglia. Cells were incubated with 50 μg/ml poly(I:C) for 6 or 24 h and then processed for RT-PCR analysis. Data are means ± SEM (*n* = 3) normalized to GAPDH levels and are representative of three experiments. **p < 0.01 and ***p < 0.001 *vs* control (‘Ctr’)

The ability of TLR ligation to influence TLR subtype expression in CNS glia remains to be explored. To address this question, we incubated purified cortical microglia with LPS, zymosan or poly(I:C) and evaluated TLR mRNA expression by RT-PCR after 6 or 24 h. LPS treatment significantly decreased or increased, respectively, TLR4 and TLR2 gene levels at both time points (Fig. 4). In contrast, TLR3 gene expression was unchanged at 6 h and significantly reduced at 24 h. Zymosan treatment of microglia likewise produced significant decreases and increases in TLR4 and TLR2 mRNA levels, respectively, at 6 and 24 h (Fig. 5), while also significantly lowering levels of TLR3 mRNA at 6 and 24 h. Engagement of TLR3 with poly(I:C) produced somewhat different results: TLR4 mRNA was decreased at 6 h only, TLR2 significantly elevated at 6 and 24 h, and no change in TLR3 gene at either time point (Fig. 6).

### TLR agonists regulate TLR subtype gene expression in purified astrocytes

**Fig. 7:**
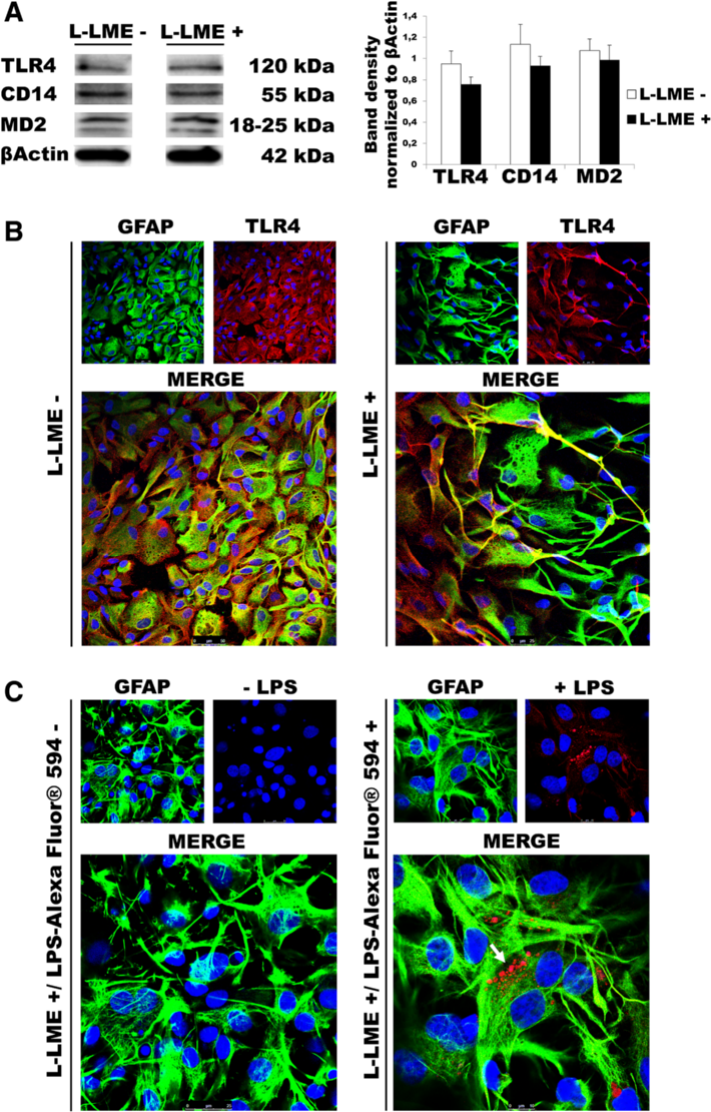
Analysis of TLR4, CD14 and MD2 expression in enriched and L-LME-purified astrocytes. **a** Western blots show comparable levels of TLR4, CD14 and MD2 in both astrocyte populations. *Left panel*: representative immunoblot. *Right panel*: Mean values from duplicate samples. **b** Confocal microscopy shows co-expression of GFAP (*green*) and TLR4 (*red*) in enriched (L-LME**-**) as well as in purified (L-LME**+**) astrocytes. *Lower frame* in each case illustrates GFAP/TLR4 overlap. Nuclei are labelled with DAPI (*blue*). **c** LPS conjugated with Alexa Fluor® 594 (*red*) co-localizes with GFAP (*green*) in L-LME-purified astrocytes after 30 min of treatment (*arrow*). Nuclei are labelled with DAPI

TLR4, together with the accessory protein MD2 and co-receptor CD14, forms a complex that binds LPS to transmit an intracellular signal [36–38]. Given the lack of responsiveness of purified astrocytes to TLR4 ligation in terms of pro-inflammatory cytokine induction, we asked whether or not these cells express the relevant receptor components. Western blot analysis using specific antibodies against TLR4, CD14 and MD2 demonstrated comparable levels of protein expression between enriched and purified astrocytes (Fig. 7a). Given that the enriched astrocytes contain a small number of microglia (3–4 % on average), it is not unexpected that removal of this component by L-LME would result in a visibly different level of component protein expression on Western blot. Indeed, lysates from an equivalent number of microglia (10,000) to be expected in 250,000 enriched astrocytes, subjected to Western blot analysis for TLR2, TLR3 and TLR4 failed to evidence a detectable signal (Additional file 1: Figure S3). Confocal microscopy revealed co-expression of GFAP and TLR4 in astrocyte-enriched as well as in L-LME-purified astrocytes (Fig. 7b). Moreover, a fluorescent conjugate of LPS was utilized to show that LPS undergoes binding and transport in astrocytes after a 30-min incubation and co-localizes with GFAP (Fig. 7c).

**Fig. 8:**
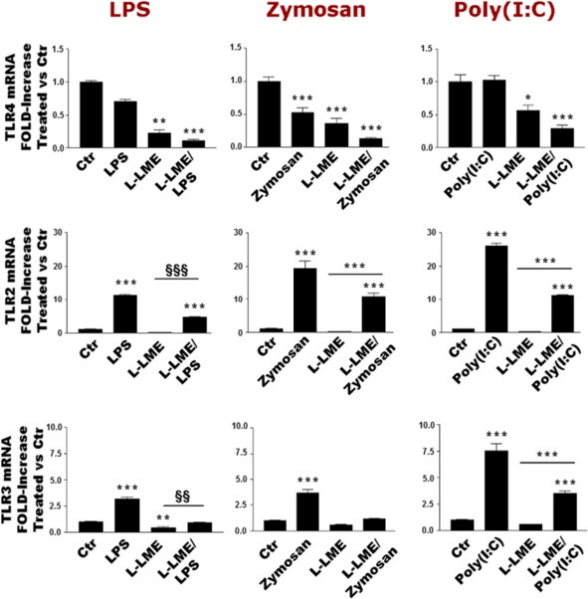
TLR agonists regulate TLR2, TLR3 and TLR4 gene expression in rat cortical astrocytes. Enriched or purified (L-LME-treated) astrocytes were challenged with LPS (100 ng/ml), zymosan (10 μg/ml) or poly(I:C) (50 μg/ml) and processed after 6 h for TLR2, TLR3 and TLR4 mRNA expression by RT-PCR. Data are means ± SEM (*n* = 3) normalized to GAPDH levels and are representative of three experiments. *p < 0.05, **p < 0.01 and ***p < 0.001 vs control (‘Ctr’). ^§§^ *p* < 0.01 and ^§§§^ *p* < 0.001 vs L-LME-treated cells. Qualitatively similar results were obtained after 24 h, although relative mRNA levels were lower

Given the general lack of responsiveness of purified astrocytes to TLR ligands in terms of pro-inflammatory cytokine induction, we then asked whether or not these cells possessed the capability to react to TLR ligation at the level of TLR expression. Enriched and L-LME-purified astrocytes were challenged with LPS, zymosan or poly(I:C) and processed after 6 h for RT-PCR analysis of TLR gene expression. LPS significantly diminished TLR4 mRNA in both astrocyte populations, while increasing expression levels of TLR2 and TLR3 (Fig. 8, left column). Likewise, zymosan and poly(I:C) each reduced the TLR4 mRNA level, while raising TLR2 and TLR3 mRNA (Fig. 8, middle and right columns, respectively). Qualitatively similar trends were observed at 24 h (data not shown). In all cases, mRNA levels were lower in purified astrocytes, possibly indicative of removal of the residual microglia subset. However, this explanation alone appears insufficient to fully account for these responses as, for example, the increase in TLR3 mRNA induced by all three TLR ligands is at variance with the lack of change in LPS- and poly(I:C)-treated microglia (Figs. 4, 5 and 6). These last findings suggest that either astrocytes are more responsive in the presence of microglia or the small numbers of contaminating microglia exhibit altered behaviours in the presence of astrocytes [7, 8, 25, 29].

### TLR agonists regulate TLR subtype levels in purified cortical microglia and astrocytes

**Fig. 9:**
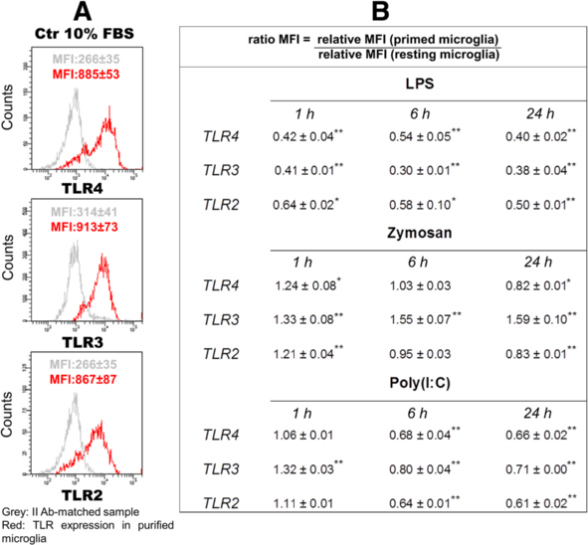
TLR ligands influence expression of their receptors in purified microglia. Microglia were challenged with LPS (100 ng/ml), zymosan (10 μg/ml) or poly(I:C) (50 μg/ml) for 1, 6 and 24 h then processed for flow cytometric analysis, as described in ‘Methods’. Based on MFI values of each TLR and corresponding II AB-matched negative control from resting (**a**) and primed cells, we compared to each other the relative MFI values [MFI TLR expression/MFI II AB-matched sample] to define ratio values [relative MFI (primed cells)/relative MFI (resting cells)] (**b**) as indicators of TLR expression change induced by treatment. Data are mean ± SD of three independent experiments conducted in duplicate. *p < 0.05 and **p < 0.01 vs control

**Fig. 10:**
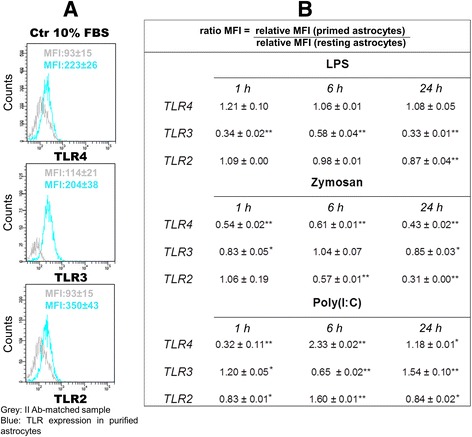
TLR ligands influence expression of their receptors in purified astrocytes. Purified (L-LME-treated) astrocytes were challenged with LPS (100 ng/ml), zymosan (10 μg/ml) or poly(I:C) (50 μg/ml) for 1, 6 and 24 h then processed for flow cytometric analysis, as described in ‘Methods’. Based on MFI values of each TLR and corresponding II AB-matched negative control detected in resting (**a**) and primed cells, we compared the relative MFI values [MFI TLR expression/MFI II AB-matched sample] to define ratio values [relative MFI (primed cells)/relative MFI (resting cells)] (**b**) as indicators of TLR expression change induced by treatment. Data are mean ± SD of three independent experiments conducted in duplicate. **p < 0.01 vs control

After treatment of purified microglia and astrocyte cultures with TLR agonists, the change in TLR expression level was defined by FCM using untreated cultures (Ctr 10 % FBS) as reference (Figs. 9 and 10). Based on MFI values of each TLR and corresponding II AB-matched negative control detected in resting (Figs. 9a and 10a) and primed (data not shown) cells, we compared each pair of relative MFI values to define ratio values (Figs. 9b and 10b) as indicators of TLR expression change induced by treatment. A complex pattern of regulation was observed in purified cortical microglia: LPS treatment significantly down-regulated surface expression of TLR2, TLR4 at all time points (1, 6, 24 h) examined (Fig. 9b). Zymosan caused a transient (1 h) increase in TLR2 and TLR4; the former was significantly lower at 24 h. Much like LPS, poly(I:C) treatment led to a significant reduction in all three TLRs at both 6 and 24 h, with a transient rise only in intracellular TLR3.

Astrocytes, nominally free of microglia likewise displayed a complex yet distinct set of responses to the three TLR ligands: LPS treatment did not affect TLR4 expression at any time point, while significantly down-regulating TLR3 at all times (1, 6, 24 h) and TLR2 only at 24 h (Fig. 10b). In contrast, zymosan significantly down-regulated TLR4 at all time points but TLR2 only at 24 h; intracellular TLR3 was not affected over the 24-h period. Poly(I:C) treatment variably increased expression of the TLRs at 6, 24, and 1 and 6 h, respectively, for TLR2, TLR3 and TLR4 (Fig. 10b).

One needs to keep in mind that fluorescence intensity values for control and TLR agonist-treated cells are reported on a logarithmic scale in Figs. 9 and 10. When the shift in intensity between isotype/II antibody-matched control and sample of interest is ≥4 fluorescence intensity values, the sample is considered positive. The stronger expression of all three TLRs on resting microglia was demonstrated by a MFI difference of ~600 between positive and negative samples and by a percentage of positive cells for each target marker of ≥75 %. In contrast, the expression of TLR4, TLR3 and TLR2 was lower in astrocytes, in accordance with several studies performed in vitro and in vivo on glial cells [39, 40]. The difference in MFI fluorescence intensity between target and control samples ranged from 90 to 257, and the percentage of positive cells was 90 % for TLR3 and 20 % for TLR4 and TLR2.

### NF-κB role in TLR agonist regulation of TLR subtype gene expression in purified cortical microglia and astrocytes

LPS interaction with TLR4 activates downstream mitogen-activated protein kinase (MAPK) and NF-κB signalling pathways and subsequently causes inflammatory mediator production [41–44]. To examine a possible role for these pathways in the regulation of TLR transcripts by LPS, the experiments described in Figs. 4 and 8 were repeated, using the NF-κB inhibitor Ro-106-9920. Incubation of microglia with 1 μM Ro-106-9920 reduced, by almost 90 %, the LPS-induced increases in TNF-α mRNA and TNF-α release after 6 h (Additional file 1: Figure S2); Ro-106-9920 (10 μM) also reduced, to a similar extent, the LPS-mediated rise in TNF-α gene expression in purified astrocytes (data not shown).

**Fig. 11:**
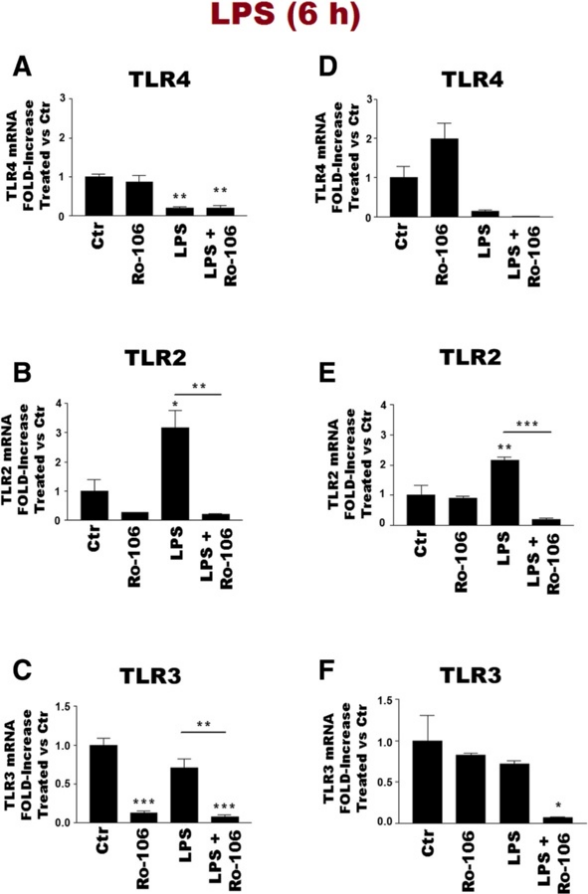
The NF-κB inhibitor Ro-106-9920 alters TLR gene expression rat cortical microglia. Cells were first incubated 30 min with 1 μM (**a–c**) or 3 μM (**d–f**) Ro-106-9920 (‘Ro-106’) in serum-free and serum-containing medium, respectively, followed by addition of LPS to a final concentration of 100 ng/ml LPS. After a further 6 h, cells were collected and processed for RT-PCR analysis. TLR4 (**a**, **d**); TLR2 (**b**, **e**); TLR3 (**c**, **f**). Data are means ± SEM (*n* = 3) normalized to GAPDH levels. **a** **p < 0.01 for LPS vs Ctr and LPS + Ro-106-9920 *vs* Ctr; **b** *p < 0.05 for LPS vs Ctr and ^§§^ *p* < 0.01 for LPS vs Ro-106-9920; **c** ***p < 0.001 for Ro-106-9920 vs Ctr and LPS + Ro-106-9920 vs Ctr, and ^§§^ *p* < 0.01 for LPS *vs* LPS + Ro-106-9920; **e** **p < 0.01 for LPS vs Ctr and ^§§§^ *p* < 0.001 for LPS vs LPS + Ro-106-9920; **f** *p < 0.05 for LPS + Ro-106-9920 vs Ctr. Similar results were obtained in a second experiment

**Fig. 12:**
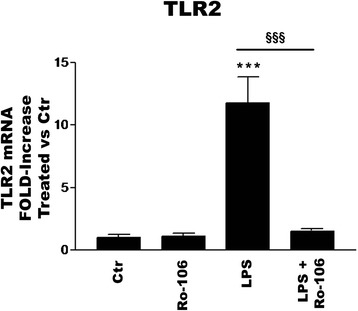
The NF-κB inhibitor Ro-106-9920 blocks LPS-induced up-regulation of TLR2 gene expression in rat cortical astrocytes. Purified (L-LME-treated) astrocytes were first incubated 30 min with 10 μM Ro-106-9920 (‘Ro-106’) in serum-containing medium, followed by addition of LPS to a final concentration of 100 ng/ml LPS. After a further 6 h, cells were collected and processed for TLR2 mRNA expression by RT-PCR. Data are means ± SEM (*n* = 3) normalized to GAPDH levels. ***p < 0.001 for LPS vs Ctr; ^§§§^ *p* < 0.001 for LPS vs LPS + Ro-106-9920. Similar results were obtained in a second experiment

NF-κB appeared to play a role in microglial TLR gene expression, as Ro-106-9920 selectively influenced TLR mRNA levels in the absence and presence of ligand (Fig. 11). Because microglia vitality was sensitive to prolonged incubation with Ro-106-9920 above 3 μM in serum-containing medium, the experiments were carried out both in serum-containing (panels a–c) and serum-free (panels d–f) media. Incubation with LPS for 6 h strongly reduced TLR4 mRNA levels in microglia (Fig. 11a, d; see also Fig. 4); Ro-106-9920 neither altered this effect nor influenced basal TLR4 gene expression. In contrast, the NF-κB inhibitor markedly inhibited (≥90 %) the LPS-induced rise in TLR2 mRNA (Fig. 11b, e); basal TLR2 gene expression was not significantly changed. In the case of TLR3, LPS did not influence mRNA levels per se at 6 h (see also Fig. 4), although incubation with Ro-106-9920 produced a robust and significant drop in TLR3 gene expression (Fig. 9c, f). Overall, microglial cell behaviours were qualitatively similar independent of the presence of serum. Ro-106-9920 likewise blocked, by more than 85 %, the LPS-induced increase in TLR2 mRNA in purified astrocytes at 6 h (Fig. 12). However, Ro-106-9920 did not effect any consistent alterations in either TLR3 or TLR4 mRNA levels in astrocytes, irrespective of stimulation with LPS (data not shown).

## Discussion

Toll-like receptors play a fundamental role in recognizing pathogens and initiating an innate immune response to protect the host. However, sterile inflammation can result when TLRs, in the process of detecting tissue damage bind endogenous ligands released by stressed or injured cells [45]. Not only immune system-related cells but also CNS neuronal and non-neuronal cell types (including microglia and astrocytes) express and respond to TLR ligation [15, 18, 45, 46]. For microglia, these behaviours include the output of pro-inflammatory cytokines following stimulation with TLR2/3/4 agonists ([7, 8, 18, 47], among others). The present study describes a novel response of microglia to TLR ligands, namely, the ability of these agents to induce homologous and heterologous changes in both TLR gene expression and cell surface TLR2/4 or intracellular TLR3 expression. Further, nominally microglia-free astrocytes, which do not respond to TLR agonists in terms of pro-inflammatory mediator production [7, 8, 25], remained responsive to TLR ligand challenge in terms of altered TLR alterations, as for microglia.

TLR activation is receiving increasing attention as being implicated in neurodegenerative disorders [19, 20], as well as in inflammatory pain and neuropathic pain [21–24, 46, 48]. Primary cultures of CNS-derived microglia and astrocytes have found widespread application for investigating the molecular events underlying TLR actions. However, the cell preparative methodologies currently used, while capable of yielding quite pure populations of microglia, result in astrocytes which generally contain a minor subset (up to 5 %) of contaminating microglia [26, 28, 49, 50]. A number of recent studies have highlighted the need for caution in interpreting observations made with such astrocyte cultures [7, 8, 25, 28, 29]. Our observations confirm that L-LME-mediated eradication of the residual microglia effectively negated astrocyte responses to TLR2/3/4 ligands in terms of IL-1β, IL-6 and TNF-α gene induction. Yet, purified astrocytes appeared to express TLR4, CD14 and MD2 by Western blot, and confocal microscopy revealed co-expression of GFAP and TLR4 in these cells. Moreover, fluorescently conjugated LPS underwent binding and transport in astrocytes and co-localized with GFAP. Thus, astrocytes have the capability to recognize this TLR4 ligand.

The observation that TLR agonists are capable of influencing gene expression not only for their cognate receptor but also for other TLRs, in both microglia and astrocytes, was somewhat unexpected. The TLR2/3/4 agonists examined (LPS, zymosan and poly(I:C), respectively) decreased TLR2 and TLR4 mRNA, while LPS and zymosan (but not poly(I:C) decreased TLR3 transcript levels at 24 h only. Apart from a report describing that cell wall components derived from Gram-negative bacteria induce TLR2 gene in the CNS [51], other studies dealing with TLR ligand effects on cognate receptor expression have generally been carried out in non-neural cell types, such as human aortic smooth muscle cells [52], cardiomyoblasts [53] and dendritic cells [54] and TLR2. In Laflamme et al. [51], peptidoglycan, a TLR2 ligand derived from Gram-negative bacteria failed to alter TLR2 expression. Olson and Miller [18] observed an increased expression of TLR2 and TLR4 (but not TLR3) mRNA in LPS (5 μg/ml)-treated mouse microglia after 7 h. Several other reports have examined the effects of TLR ligands on TLR expression in glia, although differences are to be found with the present data. Such differences include the use of astrocytes less pure than ours [39, 55, 56], lack of comparison with microglia [56], the use of a microglial cell line instead of primary cells [57], lack of analysis by flow cytometry [56, 57] and use of LPS only and not other TLR ligands [56]. As such, even a small percentage of contaminating microglia could account for the expression of TLRs observed in astrocyte cultures. Further, it is well-documented that established protocols for isolating LPS result in the co-purification of varying amounts of endotoxin protein(s) such as lipoproteins [58, 59] (addressed at length in [58] and references within). As these contaminants possess potent bioactivity, assigning cellular responses to the LPS component of a particular preparation may be confounded by the presence of these contaminants which could be responsible for the TLR2-mediated signalling observed upon LPS stimulation. The relatively high concentrations (5–25 μg/ml) of LPS used in several of the above studies [55, 56], together with a lack of information as to LPS purity needs to be considered when interpreting the data. We also performed an experiment comparing the biological activity LPS from *E. coli* 026:B6 (Sigma) with LPS-EB Ultra-Pure (InvivoGen) in the presence of polymyxin B, which competes with LPS for binding to (and activation of) TLR4. LPS (InvivoGen) activity in terms of IL-1β release and nitric oxide production from rat cortical microglia, used at 1000 ng/ml, was fully blocked by polymyxin B—in contrast to LPS from Sigma (Additional file 1: Figure S4). Therefore, the Sigma LPS contains LPS-independent activity.

The endogenous TLR4 ligand myeloid-related protein 8 induced IL-1β in astrocytes, although cells were only 95 % pure [60]. Surface expression of TLR2 and TLR4, and intracellular expression of TLR3, was consistently reduced in microglia by LPS and poly(I:C). On the other hand, changes induced by zymosan were more variable. Astrocyte responses to LPS, zymosan and poly(I:C) were substantially like those of microglia, with all three agonists down-regulating TLR4 transcripts, while up-regulating TLR2. In contradistinction to microglia, these TLR ligands up-regulated TLR3 mRNA in astrocytes. In general, the relative levels of TLR mRNA were lower in purified (L-LME-treated) astrocytes. While the latter observation is likely to reflect removal of contaminating microglia, the behaviour of TLR3 mRNA in astrocytes contrasts with a lack of change in this TLR for LPS- and poly(I:C)-treated microglia. Conceivably, astrocytes may be more responsive in the presence of microglia, or the small numbers of residual microglia could behave differently in the presence of astrocytes [7, 8, 18, 29]. Further studies will be needed to shed more light on this question. Although outside the scope of the present study, it will be interesting to explore possible developmental considerations regarding microglia and astrocyte sensitivity to TLR stimulation.

Ligand-regulated TLR expression is likely to be a complex process, especially when cross-TLR expression is involved. Indeed, changes in TLR expression, as determined by qRT-PCR and FCM did not agree with each other in several instances. A discrepancy between qPCR and corresponding protein expression has already been reported [39] and could be justified considering two different regulatory levels of TLR signalling. On the one hand, activation of MyD88-dependent and MyD88-independent pathways after agonist stimulation promotes increased expression of TLR target genes [39, 61]. Up-regulation of TLR2 mRNA is considered consequent to the activation of pro-inflammatory signalling [62], as TLR2 is a target gene of NF-κB. On the other hand, the level of TLR membrane expression is subject to regulation in order to tune cellular responsiveness to bacterial, fungal and viral insults and to reduce/increase the activation of microglia and astrocytes. Such expression may differ for a given cell type as a function of species, maturation and activation state, and is known to be modified by positive and negative feedback mechanisms [39, 63], inhibition of translational machinery [64], negative regulation by pro-inflammatory cytokines [65] and endocytic pathways [66]. After bacterial insults, astrocytes and microglia are spared from hypersensitivity through LPS-mediated endocytosis of TLRs or negative regulatory mechanisms [63]. In L-LME-treated astrocytes stimulated with Ultra-Pure LPS, membrane TLR2 expression was unchanged from 1 to 6 h but down-regulated at 24 h. In contrast, TLR4 expression was unaffected. Prior exposure to LPS reportedly induces a transient state of endotoxin tolerance (cell refractoriness) to LPS re-stimulation which correlates with reduced cell surface expression of the LPS receptor complex (TLR4/MD-2) or with other inhibitory mechanisms downstream of TLR4/MD-2. In the present study, analysis of TLRs on glial cells suggested that purified astrocytes retain their surveillance activity against a specific stimulus without changing expression of the related TLR (i.e. TLR4 for astrocytes stimulated with LPS), but rather induce tolerance to other stimuli by decreasing expression of their corresponding receptor.

It is important to keep in mind that the aim of this study was to define cell responsiveness to TLR ligands; thus, we used flow cytometry analysis on non-permabilized samples to detect plasma membrane-exposed TLR2 and TLR4. Different types of information are derived from FCM, immunofluorescence and Western blot. FCM permits one to quantify the expression of target proteins using a defined cell number, whereas immunofluorescence allows for the detection of TLR4 distribution together with GFAP in one region of interest of limited area. Moreover, Western blot analysis was performed using 10 μg total proteins from each sample (corresponding to 250,000 cells—far greater than the 10,000 cells routinely used for FCM analysis). In order to detect and better quantify minimal changes in the expression of TLRs upon treatment with LPS, poly(I:C) or zymosan, FCM was preferred to Western blotting because the former is a rapid and reliable method offering high sensitivity of detection compared to alternative methods [67]. Due to this higher sensitivity in detecting minimal differences among samples and cells of heterogenous samples, FCM is used instead of Western blotting at the clinical level as an elective method for microbiological studies [68], immunophenotyping of haematological disorders [69] and monitoring of HIV-infected patients [70]. Moreover, in several studies on TLR signalling during the immune response of microglia and astrocytes performed using FCM combined with qPCR, FCM data were not confirmed by Western blot [39, 55, 71], perhaps as a consequence of differences in sample preparation which maintains conformational epitope(s) in FCM but results in its destruction in Western blot. Flow cytometry as well allows one to analyse target markers considering a defined number of cells and to correlate the expression of TLRs with the physical parameters (cell size and complexity) of each glial cell subset.

Activation of the p38 MAPK/NF-κB signalling cascade by ligand engagement of TLR2 and TLR4 is central to the production of pro-inflammatory cytokines such as TNF-α and a number of interleukin family members. Interestingly, an established NF-κB inhibitor was efficacious in blocking LPS-induced up-regulation of TLR2 mRNA in both purified microglia and astrocytes. The p65 subunit of NF-κB is a substrate for p38 [72], and the p38 inhibitor SB-202190 was also efficacious in substantially blocking the LPS effects on TLR2 (and TNF-α) gene expression in both cell populations (Marinelli and Skaper, unpublished observations). A more complete elucidation of the molecular components involved in this action awaits further studies.

TLR2 and TLR4 activation in particular, in concert with microglia and astrocytes, comprise key elements in the initiation and maintenance of neuropathic pain [48, 73–76]. The finding that both homologous (zymosan) and heterologous (LPS, poly(I:C)) TLR ligands are capable of regulating TLR2 gene expression may have important implications in understanding the relative contributions of different TLRs in neurological disorders associated with neuroinflammation.

## Conclusions

The present study is the first to examine the regulation of various TLR subtype genes as a consequence of TLR signalling in purified microglia and astrocytes, two cell types implicated in the initiation and maintenance of neuroinflammation. In particular, our findings have isolated independent contributions of TLR stimulation on astrocytes and microglia. These observations may have important implications in understanding the participation of different TLRs in pain and other neurological disorders.

## Additional file 1

Additional file 1:
**Figure S1–S4.** Figure S1 l-leucyl-l-leucine methyl ester (L-LME) depletes enriched astrocytes of microglia. Following mechanical separation of microglia from the mixed glial cell monolayers, astrocytes were detached, replated, and incubated the next day with 50 mM L-LME for 60 min. Cells were returned to fresh culture medium and processed 24 h later for Iba1 mRNA by RT-PCR. L-LME- treated astrocytes displayed an Iba1 mRNA level of 0.17-fold difference compared to enriched (≥95 %) astrocytes. Parallel sets of cultures were processed for GFAP (red) and Iba1 (green) immunocytochemistry (lower panels). Nuclei are labelled blue with DAPI. Note the loss of residual microglia (arrows) in the L-LME-treated culture. Figure S2 The NF-κB inhibitor Ro-106-9920 blocks LPS-induced TNF-α gene and protein expression in purified rat cortical microglia. Cells were pretreated 30 min with 1 μM Ro-106-9920 (‘Ro-106’), followed by addition of LPS (100 ng/ml final) and incubation continued for a further 6 h. Cells were then collected and processed for mRNA analysis by RT-PCR (left panel) and culture medium for TNF-α content by ELISA (right panel). Values are means ± s.e.m. (n = 3). ****p* < 0.001 for LPS *vs* Ctr or Ro-106-9920; ^§§§^ *p* < 0.001 for LPS *vs* LPS + Ro-106-9920. Figure S3 Western blot analysis of TLR2, TLR3 and TLR4 expression in purified rat cortical microglia. Cell lysates were prepared from 10,000 cells and probed for the indicated TLR as described in Methods. This number of microglia corresponds to their expected contribution in 250,000 enriched astrocytes analysed in Fig. 7a. As can be seen. This number of microglia is insufficient to produce a signal. Figure S4 Effect of polymyxin B on nitric oxide production and IL-1β release from rat cortical microglia stimulated with different commercial sources of LPS. Purified microglia were challenged with LPS (1 μg/ml) obtained from Sigma (<5 % protein impurities) or InivoGen (LPS-EB Ultra-Pure). Where indicated, the LPS antagonist polymyxin B was included in the culture (10 μg/ml). Cell culture medium was collected after 24 h and processed for IL-1β content by ELISA. Data are means ± SEM (n = 3). Note the lack of cell responsiveness to LPS-EB (but not LPS from Sigma) in the presence of polymyxin B. (DOC 3981 kb)

## Abbreviations

DMEM: Dulbecco’s modified Eagle’s medium
FCS: fetal calf serum
GFAP: glial fibrillary acidic protein
Iba1: ionized calcium-binding adapter molecule 1
IL-1β: interleukin-1β
L-LME: l-leucyl-l-leucine methyl ester
LPS: lipopolysaccaride
MAPK: mitogen-activated protein kinase
MFI: mean fluorescent intensity
NF-κB: nuclear factor-κB
Poly(I:C): polyinosinic-polycytidylic acid
qRT-PCR: quantitative Real-Time-PCR
TLR: Toll-like receptor
TNF-α: tumor necrosis factor-α
