# Supplementary material for: Ligand engagement of Toll-like receptors regulates their expression in cortical microglia and astrocytes
Source: J Neuroinflammation. 2015 Dec 30;12:244. doi: 10.1186/s12974-015-0458-6 (PMC4696218; doi:10.1186/s12974-015-0458-6)
Supplement: Additional file 1: — Figure S1–S4. Figure S1 l-leucyl-l-leucine methyl ester (L-LME) depletes enriched astrocytes of microglia. Following mechanical separation of microglia from the mixed glial cell monolayers, astrocytes were detached, replated, and incubated the next day with 50 mM L-LME for 60 min. Cells were returned to fresh culture medium and processed 24 h later for Iba1 mRNA by RT-PCR. L-LME- treated astrocytes displayed an Iba1 mRNA level of 0.17-fold difference compared to enriched (≥95 %) astrocytes. Parallel sets of cultures were processed for GFAP (red) and Iba1 (green) immunocytochemistry (lower panels). Nuclei are labelled blue with DAPI. Note the loss of residual microglia (arrows) in the L-LME-treated culture. Figure S2 The NF-κB inhibitor Ro-106-9920 blocks LPS-induced TNF-α gene and protein expression in purified rat cortical microglia. Cells were pretreated 30 min with 1 μM Ro-106-9920 (‘Ro-106’), followed by addition of LPS (100 ng/ml final) and incubation continued for a further 6 h. Cells were then collected and processed for mRNA analysis by RT-PCR (left panel) and culture medium for TNF-α content by ELISA (right panel). Values are means ± s.e.m. (n = 3). ***p < 0.001 for LPS vs Ctr or Ro-106-9920; §§§ p < 0.001 for LPS vs LPS + Ro-106-9920. Figure S3 Western blot analysis of TLR2, TLR3 and TLR4 expression in purified rat cortical microglia. Cell lysates were prepared from 10,000 cells and probed for the indicated TLR as described in Methods. This number of microglia corresponds to their expected contribution in 250,000 enriched astrocytes analysed in Fig. 7a. As can be seen. This number of microglia is insufficient to produce a signal. Figure S4 Effect of polymyxin B on nitric oxide production and IL-1β release from rat cortical microglia stimulated with different commercial sources of LPS. Purified microglia were challenged with LPS (1 μg/ml) obtained from Sigma (<5 % protein impurities) or InivoGen (LPS-EB Ultra-Pure). Where indicated, the LPS antagonist polym [file 12974_2015_458_MOESM1_ESM.doc]

**Supplementary Information**

**Ligand engagement of Toll-like receptors regulates their expression in cortical microglia and astrocytes**

Carla Marinelli, Rosa Di Liddo, Laura Facci, Thomas Bertalot, Maria Teresa Conconi, Morena Zusso, Stephen D. Skaper and Pietro Giusti


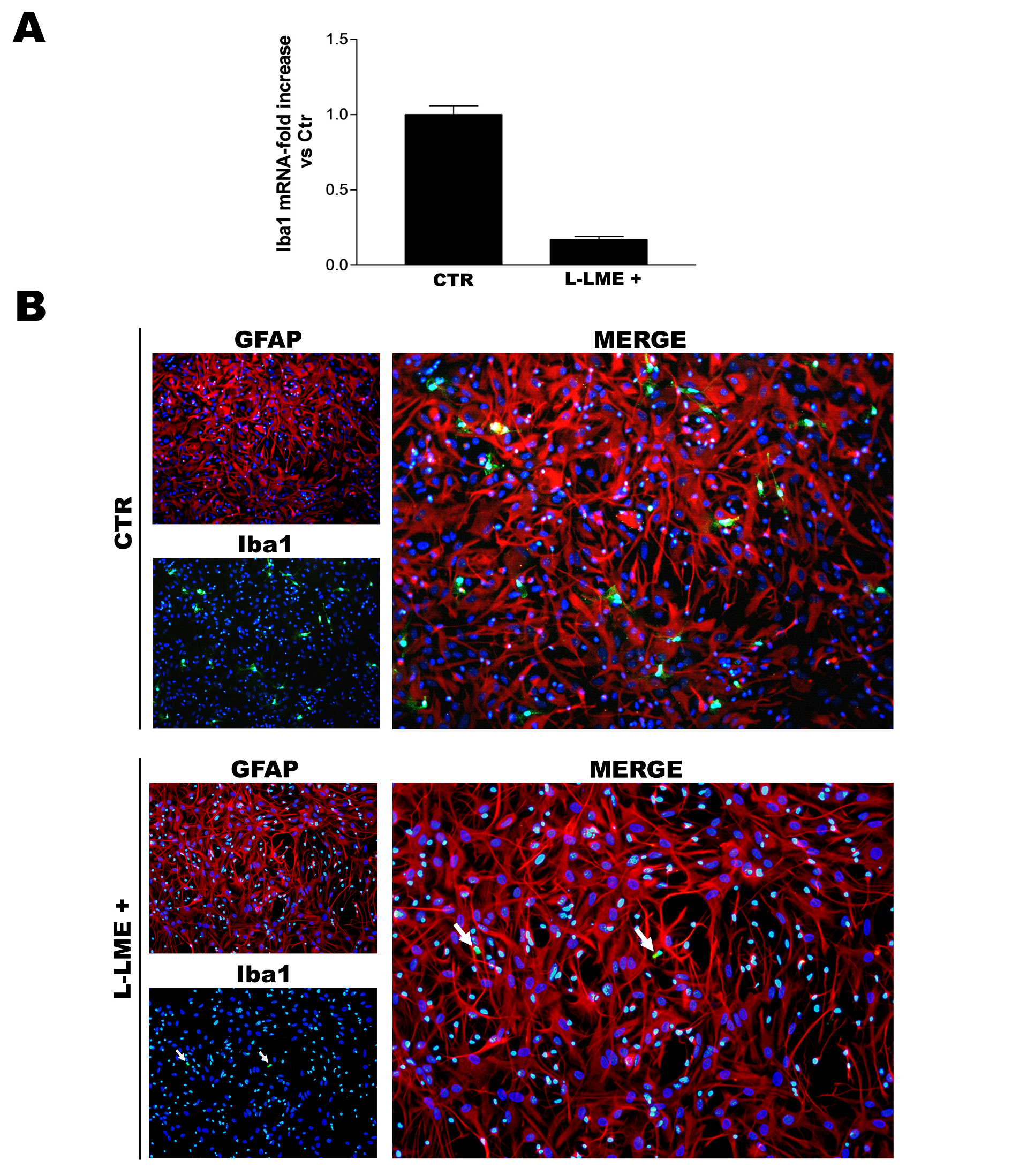


**Supplementary Figure S1.** L-leucyl-L-leucine methyl ester (L-LME) depletes enriched astrocytes of microglia. Following mechanical separation of microglia from the mixed glial cell monolayers, astrocytes were detached, replated, and incubated the next day with 50 mM L-LME for 60 min. Cells were returned to fresh culture medium and processed 24 h later for Iba1 mRNA by RT-PCR. L-LME- treated astrocytes displayed an Iba1 mRNA level of 0.17-fold difference compared to enriched (≥95%) astrocytes.

Parallel sets of cultures were processed for GFAP (red) and Iba1 (green) immunocytochemistry (lower panels). Nuclei are labelled blue with DAPI. Note the loss of residual microglia (arrows) in the L-LME-treated culture.


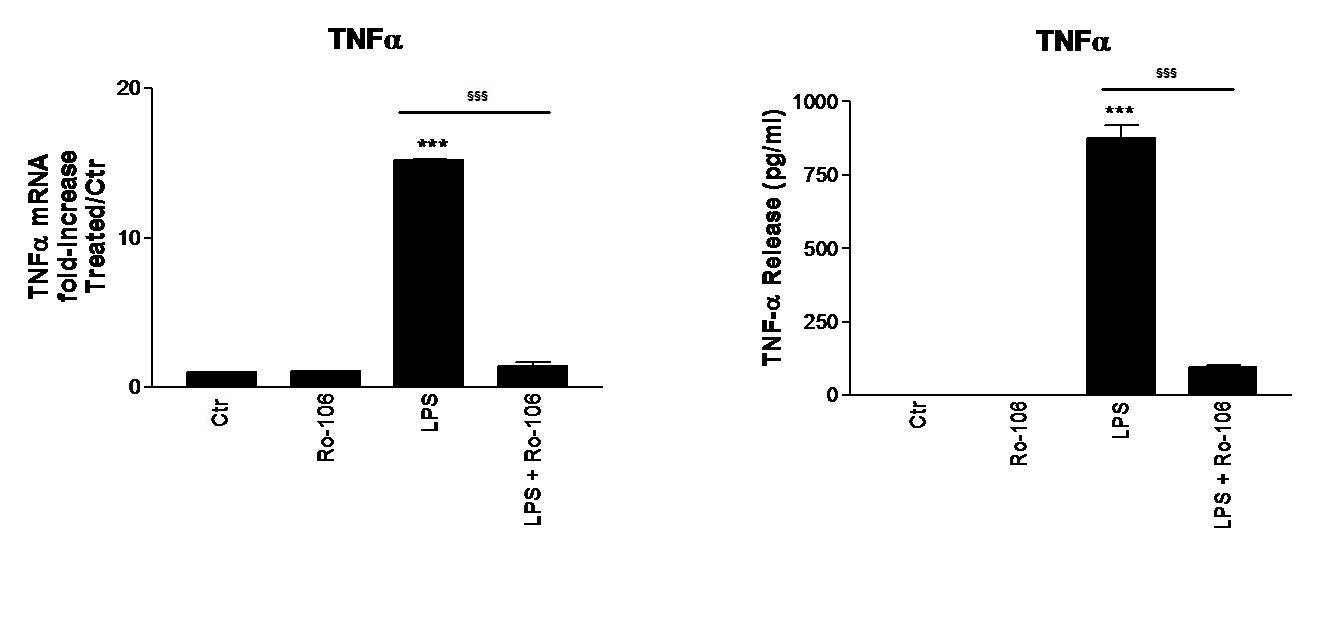


**Supplementary Figure S2.** The NF-κB inhibitor Ro-106-9920 blocks LPS-induced TNF-α gene and protein expression in purified rat cortical microglia. Cells were pretreated 30 min with 1 μM Ro-106-9920 ('Ro-106'), followed by addition of LPS (100 ng/ml final) and incubation continued for a further 6 h. Cells were then collected and processed for mRNA analysis by RT-PCR (left panel) and culture medium for TNF-α content by ELISA (right panel). Values are means + s.e.m. (n=3). ****p*<0.001 for LPS *vs* Ctr or Ro-106-9920; ^§§§^*p*<0.001 for LPS *vs* LPS + Ro-106-9920.


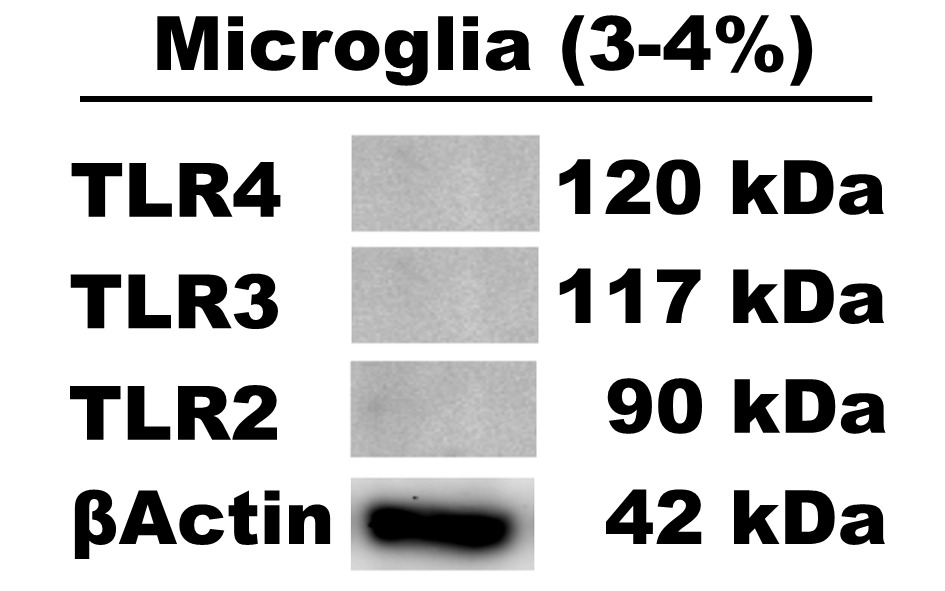


**Supplementary Figure S3.**  Western blot analysis of TLR2, TLR3 and TLR4 expression in purified rat cortical microglia. Cell lysates were prepared from 10,000 cells and probed for the indicated TLR as described in Methods. This number of microglia corresponds to their expected contribution in 250,000 enriched astrocytes analyzed in Figure 7A. As can be seen. This number of microglia is insufficient to produce a signal.

**Supplementary Figure S4.** Effect of polymyxin B on nitric oxide production and IL-1β release from rat cortical microglia stimulated with different commercial sources of LPS. Purified microglia were challenged with LPS (1 µg/ml) obtained from Sigma (<5% protein impurities) or InivoGen (LPS-EB Ultra Pure). Where indicated, the LPS antagonist polymyxin B was included in the culture (10 µg/ml). Cell culture medium was collected after 24 h and processed for IL-1β content by ELISA. Data are means ± SEM (n=3). Note the lack of cell responsiveness to LPS-EB (but not LPS from Sigma) in the presence of polymyxin B.
